# A novel anterior nasal swab to detect respiratory viruses: a prospective study of diagnostic accuracy

**DOI:** 10.1186/s12887-023-03976-5

**Published:** 2023-04-28

**Authors:** Shidan Tosif, Lai-yang Lee, Jill Nguyen, Isabella Overmars, Chris Selman, Anneke C. Grobler, Alissa McMinn, Gregory Waller, Sarah McNab, Tayla Jarvis, Andrew Steer, Franz E. Babl, Andrew Daley, Nigel W. Crawford

**Affiliations:** 1 Department of General Medicine, The Royal Children’s Hospital Melbourne, Melbourne, Australia; 2 Infection and Immunity, Murdoch Children’s Research Institute, 50 Flemington Rd, Melbourne, VIC 3052 Australia; 3 Department of Paediatrics, The University of Melbourne, Melbourne, Australia; 4 Department of Microbiology, The Royal Children’s Hospital Melbourne, Melbourne, Australia; 5 Clinical Epidemiology & Biostatistics Unit, Murdoch Children’s Research Institute, Melbourne, Australia; 6 Emergency Department, The Royal Children’s Hospital Melbourne, Melbourne, Australia

**Keywords:** Respiratory viral testing, Respiratory virus, Procedural anxiety, Pediatrics

## Abstract

Detection of respiratory viruses requires testing of the upper respiratory tract to obtain specimens for analysis. However, nasal and throat swabs can cause discomfort and procedural anxiety in children. Respiratory sampling methods which are accurate and less invasive are needed. We aim to determine the positive and negative percentage agreement of a novel anterior nasal swab (ANS) compared with the combined throat and anterior nasal swab (CTN), the reference standard, for detection of respiratory viruses. Children 5 – 18 years of age presenting to a tertiary paediatric hospital with respiratory symptoms were tested with both swabs in randomised order. Respiratory samples were tested on a multiplex RT-PCR panel. Viral detections, RT-PCR cycle-threshold values and child/parent/clinician experience of the swab were recorded. There were 157 viral detections from 249 participant CTN swabs. In comparison with the CTN, the overall positive and negative percentage agreement of ANS for detection of respiratory viruses was 96.2% (95% CI, 91.8–98.3%) and 99.8% (95% CI, 99.6–99.9%), respectively. The ANS was “extremely comfortable”, or only a “little uncomfortable” for 90% of children compared with 48% for CTN. 202 children (84%) rated the ANS as the preferred swab, and 208 (87%) indicated they would prefer ANS for future testing. The ANS required additional laboratory handling processes compared to the CTN. The ANS has high positive percentage agreement and is comparable to the current standard of care. The high acceptability from the less invasive ANS provides a more comfortable method for respiratory virus testing in children.

**Trial registration**

ClinicalTrials.gov ID NCT05043623.

**Supplementary Information:**

The online version contains supplementary material available at 10.1186/s12887-023-03976-5.

## Introduction

Testing for respiratory viruses in children has implications for clinical assessment, treatment, and public health surveillance. Upper respiratory sampling collected by a nasal swab is the preferred method for the accurate identification of respiratory viruses in children, including respiratory syncytial virus (RSV) [1], influenza [2] and SARS-CoV-2, by reverse transcriptase polymerase chain reaction (RT-PCR). Yet sample collection in children is challenging due to feasibility issues and often requires trained health care workers to obtain samples.

Children who require frequent procedures are specifically at risk of adverse psychological impact [3]. Procedural discomfort is a commonly cited concern by parents and may present a barrier to testing [4]. Parents and children express concern for the stress, pain or discomfort from viral testing [5]. Whilst anterior nasal swabs are feasible and more acceptable to children due to reduced discomfort, sensitivity in previous studies is less than nose and throat swabs [6-8].

A novel flocked anterior nasal swab (ANS) has been recently designed for children with the aim of reducing discomfort whilst maintaining diagnostic validity (Rhinoswab Junior, Rhinomed, Melbourne, Australia). An adult version has been used for asymptomatic SARS-CoV-2 screening in adolescents [9]. The ANS has design features which may help with distraction, accurate anatomical positioning, and self-collection by the child. We conducted a prospective study to compare the positive and negative percentage agreement of ANS with the combined throat and anterior nasal (CTN) swab for the detection of respiratory viruses among children aged 5–18 years with symptoms of respiratory tract infection. We also assessed acceptability of both swabs and preference of method for future testing.

## Methods

### Study design

The study was conducted at the Respiratory Infection Clinic at The Royal Children’s Hospital (RCH), a large tertiary paediatric hospital in Melbourne, Australia, between August and November 2021. All children had both methods of sample collection, the ANS and the standard of care CTN swab, and the order of sample collection was randomised. This study has been reported using the Standards for Reporting of Diagnostic Accuracy Studies guidelines 2015 [10].

### Participants

Symptomatic children between the ages of 5–18 years were invited to participate following informed consent by their parent or guardian. Asymptomatic children were excluded.

### Test methods

**Fig. 1:**
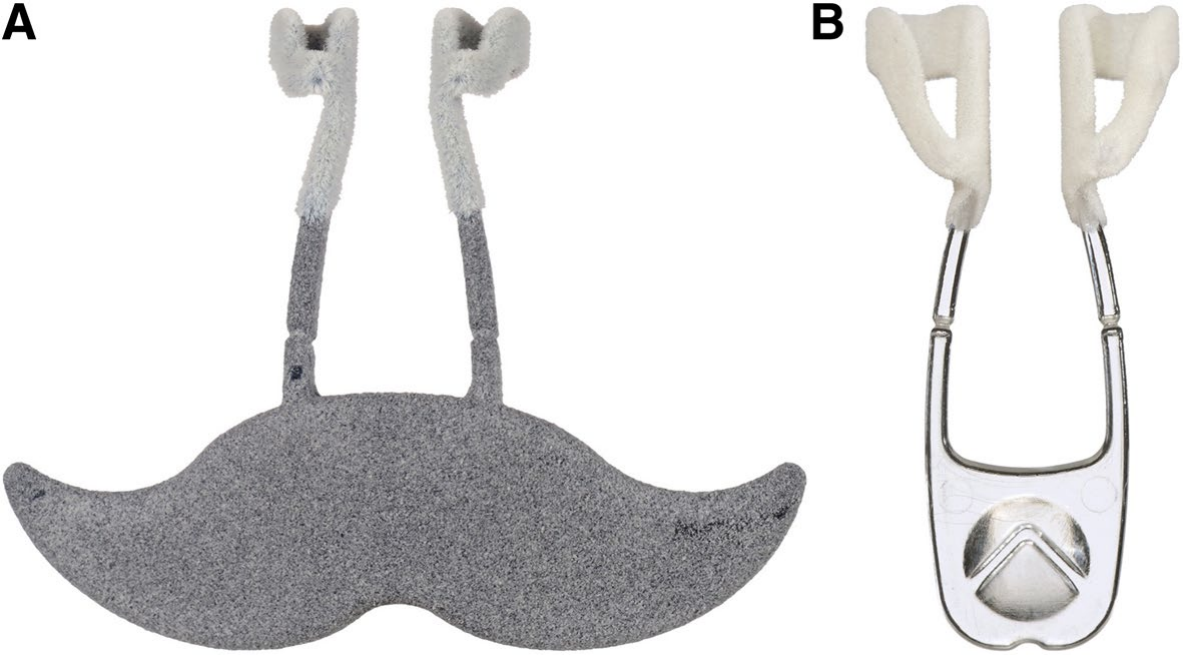
**A** Junior ANS. **B** Adult ANS

Children were considered symptomatic if they displayed any sign or symptom of a respiratory tract infection (e.g., cough, fever, sore throat). The swab order was randomised (1:1) using an online Research Electronic Data Capture platform (REDCap [11, 12]). The ANS was available in three sizes. Size selection was according to age: 5–8 years “Small”, 9–12 years “Regular” and > 12 years “Adult” (see Fig. 1A and B). The “Small” and “Regular” sizes were provided as 3-D printed clinical prototypes. The ANS was self-administered by the participant, or if assistance was needed, by the parent/guardian or study nurse. The ANS was inserted for 60 s, followed by side-to-side movements for 15 s in the anterior nasal area. The ANS swab was snapped off from the handle into a sterile closed container for transport. For CTN, the study nurse used a flocked swab (Copan Diagnostics Inc, Corona, CA) and swabbed the tonsillar beds and back of throat for 3–5 s, followed by bilateral nasal insertion and rotated 5 times against the nasal wall (1-2 cm insertion or until resistance was met). CTN swabs were placed into a sterile closed container for transport. No transport medium was used for either swab.

### Laboratory analysis

The index test was the ANS and the reference standard was the CTN swab. In the laboratory, all samples were eluted into 500ul phosphate buffered saline (PBS). ANS samples were vortexed and pulse spun while CTN samples were swirled. All samples were extracted on Roche MagNA Pure 96 system using MagNA Pure 96 DNA and Viral NA Small Volume Kit and tested on the AusDiagnostics Respiratory Pathogens 16-well assay (Mascot, Australia), on the AusDiagnostics High-Plex 24 system. The respiratory panel included SARS-CoV-2 (with SARS-CoV-2 ORF-1 and ORF-8 genes), influenza A, influenza B, respiratory syncytial virus A and B, rhinovirus, enterovirus, parechovirus, parainfluenza 1–4, adenovirus, human metapneumovirus and two bacteria, *Bordetella pertussis* and *Mycoplasma pneumoniae.* Non-SARS-CoV-2 viruses were reported as “Detected” if the cycle threshold (CT) value was less than 38.73 which is in accordance with the laboratory’s established cut off values. Samples which yielded any CT values for SARS-CoV-2 were confirmed with an alternative assay (Allplex SARS-CoV-2 Assay, Seegene, Seoul, South Korea) as per public health requirements.

### Acceptability evaluation

Acceptability was assessed by an electronic survey following the swabs. A 5-point Likert scale or Wong-Baker FACES scale [13] were used to rate comfort by the child (self-report). The parent/guardian and nurse rated the observed comfort level of the child and preference for future swabs. The person who inserted the swab (child/parent/nurse) and outcome of the ANS insertion was recorded (successful/partially successful/unable to be inserted).

### Statistical analysis

Data were collected and stored in REDCap before analysis in Stata (Version 17.0) [14]. With 95% confidence intervals (CI), the positive and negative percentage agreement of ANS were calculated for each virus and for all viruses combined. The median difference in CT values between the ANS and the standard CTN swab was compared. The CT value for the undetected sample was set at the maximum number of cycles performed in the laboratory (38.73). The following subgroups analyses were prespecified: Age (5 to 7 years, 8 to 11 years, 12 years and older), final testing order, ANS size, swab dwell time (less than 60 s, 60 s or more) and subjective impression of insertion quality by the child (good/okay/bad). Clustering analysis was used to determine confidence intervals since several pathogens could be included from the same swab/child. If the upper limit of the 95% CI for the median difference was less than 3 CT, the ANS would be regarded as non-inferior.

### Sample size calculation

Routine laboratory surveillance at our institution in the 3 months preceding the study reflected a virus was identified in 30–50% of respiratory tests. The study design included 250 participants and anticipated a minimum of 38% (*n* = 96) participants would test positive for at least one respiratory virus. With a sample size of 96, a two-sided 95% CI for positive percentage agreement would extend from 0.97 to 1, if we assume the positive percentage agreement to be 0.99, using the large sample normal approximation.

### Ethics

Informed consent was obtained from all parents/guardians before participation. Ethics approval was given by The Royal Children’s Hospital Human Research Ethics Committee (HREC 77305). This trial is registered on ClinicalTrials.gov (NCT05043623).

## Results

### Patient characteristics

Of 254 participants enrolled, 249 completed the study. Five participants were excluded as they did not have the ANS due to distress/refusal following the CTN. Ten participants (4%) had both swabs although not randomised. Median age was 6.9 years (Interquartile range (IQR) 5.1–9.9), and median number of days since symptom onset was 1 (IQR 0–2). There were 157 viral detections from 249 CTN swabs (Table 1). One hundred thirty four children had 0 viruses, 75 had 1, 38 had 2 and 2 had 3, detected on the CTN swab. 7 (3%) ANS failed to yield a result due to sample inhibition. 24 (9.6%) ANS needed to be rerun, compared with 5 (2%) CTN.

**Table 1:** Positive and negative percentage agreement of ANS compared with CTN for detection of respiratory viruses

**Pathogen**	Detected on CTN n (%)	**Result on ANS (n)**		**Positive percentage agreement (95% CI)**	**Not detected on CTN—n (%)**	**Result on ANS (n)**		**Negative percentage agreement (95% CI)**
		**Detected**	**Not detected**			**Detected**	**Not detected**	
**Influenza A**	N/A				242 (100.0%)	0	242	100.0 (98.5, 100.0)
**Influenza B**	N/A				242 (100.0%)	0	242	100.0 (98.5, 100.0)
**Respiratory Syncytial Virus**	N/A				242 (100.0%)	0	242	100.0 (98.5, 100.0)
**Rhinovirus/Enterovirus**	85 (35.1%)	83	2	97.6 (91.8, 99.7)	157 (64.9%)	0	157	100.0 (97.7, 100.0)
**Enterovirus**	35 (14.5%)	32	3	91.4 (76.9, 98.2)	207 (85.5%)	3	204	98.6 (95.8, 99.7)
**Paraechovirus**	N/A				242 (100.0%)	0	242	100.0 (98.5, 100.0)
**Parainfluenza viruses 1–3**	1 (0.4%)	0	1	0.000 (0.0, 97.5)	241 (99.6%)	0	241	100.0 (100.0, 100.0)
**Parainfluenza viruses 4**	N/A				242 (100.0%)	0	242	100.0 (98.5, 100.0)
**Adenovirus**	2 (0.8%)	2	0	100.0 (15.8, 100.0)	240 (99.2%)	1	239	99.6 (97.7, 100.0)
**Human metapneumovirus**	24 (9.9%)	24	0	100.0 (85.8, 100.0)	218 (90.1%)	1	217	99.5 (97.5, 100.0)
***Bordetella pertussis***	N/A				242 (100.0%)	0	242	100.0 (98.5, 100.0)
***Mycoplasma pneumoniae***	N/A				242 (100.0%)	0	242	100.0 (98.5, 100.0)
**SARS-CoV-2**	10 (4.1%)	10	0	100.0 (69.2, 100.0)	232 (95.9%)	0	232	100.0 (98.4, 100.0)
**All pathogens combined**	157	151	6	96.2 (91.8, 98.3)	2989	5	2984	99.8 (99.6, 99.9)

### Positive and negative percentage agreement

**Fig. 2:**
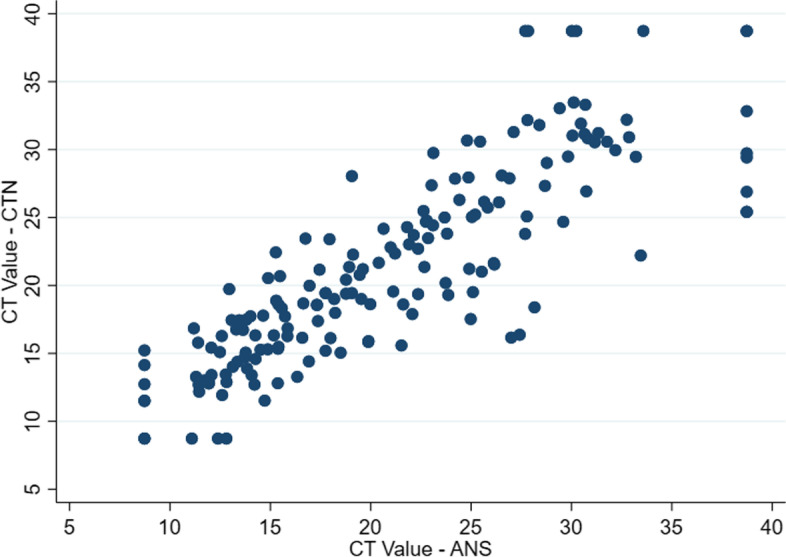
Scatterplot of ANS and CTN CT values

One hundred fifty one viruses were detected by both the CTN and ANS. Positive percentage agreement was 96.2% (95% CI, 91.8–98.3%, Table 1) with 6 detections on CTN that were not detected on ANS. Negative percentage agreement was 99.8% (95% CI, 99.6–99.9%). There were 5 detections on ANS that were not detected by CTN. Median CT value difference for all viruses combined was 0.9 lower with ANS than CTN (95% CI, 0.3–1.5). Scatterplot of CT values for CTN and ANS showed a strong positive linear association (see Fig. 2). Subgroup comparison analysis showed no difference between age, final swab sequence, ANS size, swab dwelling time or quality of insertion (Supplementary Figs. 1 and 2).

### ANS Insertion experience

One hundred forty-one (57%) participants self-inserted the ANS while 91 (37%) required nurse-assistance, and 16 (6.5%) required parent/guardian assistance. The insertion was described as “good” by 232 (93%), “okay” 16 (6%), and “bad” for one child. For 10 (4%) children, the ANS failed to fit on first attempt, and an alternative ANS size was then successfully inserted.

### Swab attitudes and preference

**Fig. 3:**
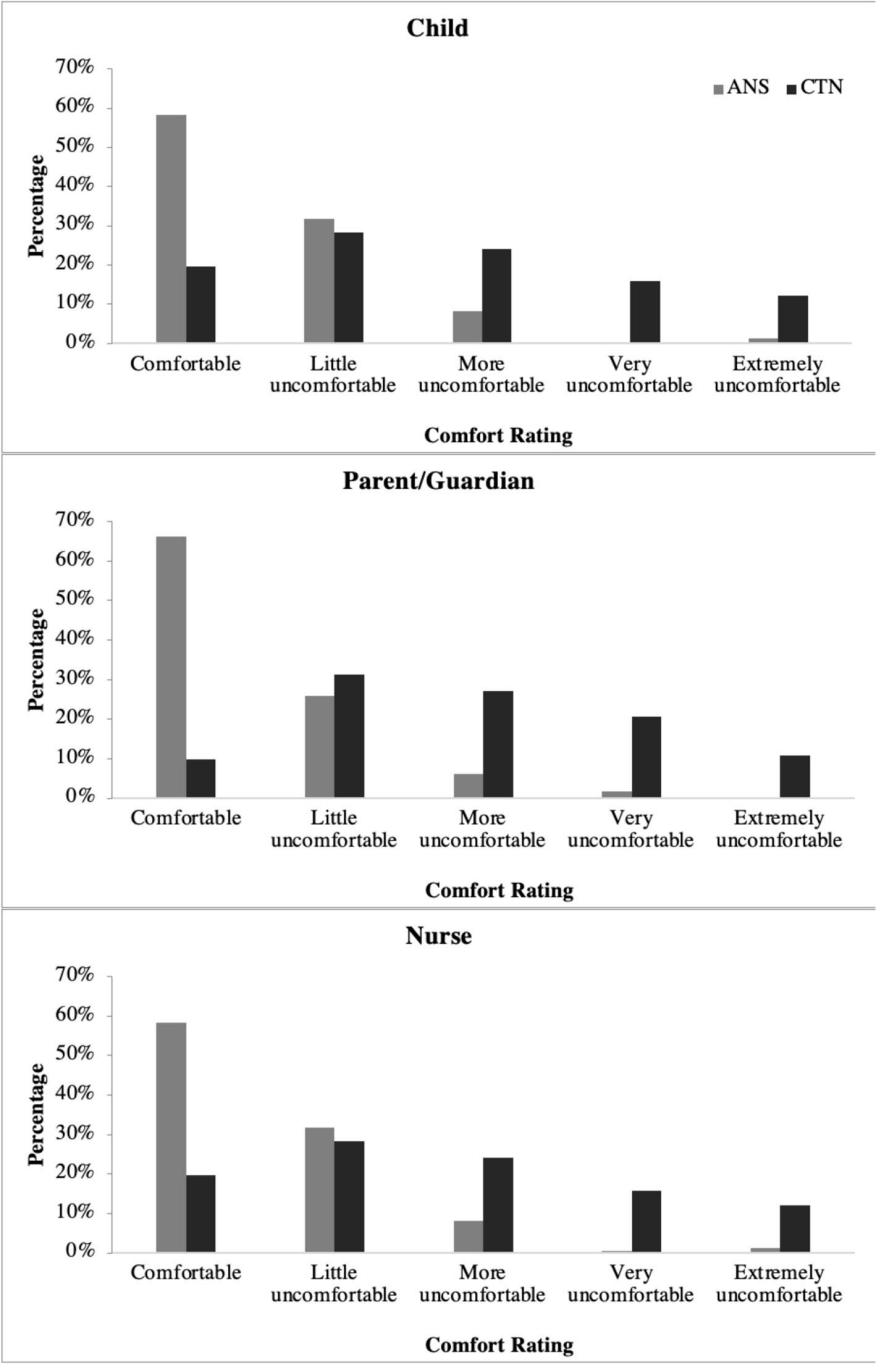
Assessment of comfort levels between ANS and standard CTN

110/239 (46%) children and 62/238 adults (26%) felt worried/nervous prior to their child receiving a respiratory test. 219/243 (90%) children, 221/240 (92%) parent/guardians, 230/248 (93%) nurses felt the ANS was “comfortable”, or a “little uncomfortable” compared with 115/240 (48%), 99/240 (41%), 87/249 (35%) for CTN respectively (Fig. 3). The majority of children, parents/guardians and nurses said the ANS was the better swab and would be preferred for future testing (see Table 2).

**Table 2:** Swab preference

	**ANS- n (%)**	**CTN—n (%)**	**No swab preferred—n (%)**
Which swab was preferred (child)?—*n* = 241	202 (84.0%)	26 (10.8%)	13 (5.4%)
If you had to have another test, which one would you choose? (child)—*n* = 240	208 (86.7%)	29 (12.1%)	3 (1.3%)
Which swab did you prefer (parent)?—*n* = 243	201 (82.7%)	12 (4.9%)	30 (12.3%)
Which swab would make you feel better about having your child tested in the future? (parent)—*n* = 242	198 (81.8%)	8 (3.3%)	36 (14.9%)
Which swab offered the better experience for the child? (nurse)—*N* = 247	209 (84.6%)	21 (8.5%)	17 (6.9%)

## Discussion

We found that the ANS had high accuracy for detection of respiratory viruses by PCR when compared with CTN (positive percentage agreement 96% and negative percentage agreement 99%). When rated by children, parents, and nurses, the ANS revealed high acceptability, with most indicating they would prefer ANS than CTN for future testing.

Selection of respiratory sampling methods in children require consideration of comfort, feasibility, diagnostic yield, time taken, and cost. Several avenues exist for respiratory viral testing including nasopharyngeal, nasal, oropharyngeal and saliva specimens. Nasal swab specimens are most frequently used in children and have high positive percentage agreement when compared to more invasive nasopharyngeal swabs [15, 16]. Collection of saliva offers a less invasive method and is relatively easy to collect, however previous studies in children, comparing nasal and nasopharyngeal swab with saliva and throat swabs, have demonstrated inferior detection for respiratory viruses [17, 18]. Adult studies investigating saliva for respiratory virus detection have described lower sensitivity and laboratory challenges due to handling of more viscous samples [19]. With the additional benefits of high accuracy and acceptability over standard testing, the ANS used in this study provides a new option for children amongst existing methods.

Large-scale testing of symptomatic and asymptomatic patients requires innovation in sampling methods and self-sampling [20]. Consideration needs to be given to respiratory testing methods that reduce procedural distress and impact from repeated procedures [3]. Frequent testing of asymptomatic children has been used to enable school attendance, or prior to elective hospital admissions in children with chronic illness throughout the SARS-CoV-2 pandemic. Some modelling has also suggested that accessibility and high frequency testing, may be a priority over test sensitivity in achieving effective population screening [21]. Studies have described lower pain scores were reported for ANS compared with combined anterior nasal and throat swabs [7, 8]. This finding is supported by our study, whereby participants and their parents reported the novel ANS caused less discomfort and was preferred over CTN. These benefits may support children to undertake more frequent testing if required and reduce procedural distress.

Methods which allow for self-collection reduce the need for clinician involvement, and associated resource and workforce requirements, personal protective equipment usage and nosocomial exposure risk. Moreover, self-collected samples for respiratory viruses have the advantage of earlier collection timed with the onset of symptoms, which may allow better detection [22]. Previous studies have highlighted potential improved uptake, higher satisfaction and reassuring diagnostic accuracy in self and caregiver collected samples [23, 24]. In this study, the median age of participants was 6.9 years, and yet 57% of participants inserted the ANS independently, highlighting the potential for self-collection in young children.

Although Australian and Victorian guidelines recommend combined anterior nasal and throat swabs for SARS- CoV-2 detection, many international guidelines accept nasal swabs alone for SARS-CoV-2 detection including the US Centers for Disease Control and Prevention [25]. A recent systematic review suggested nasal swabs are a clinically acceptable alternative specimen collection method [26]. Our data supports the use of anterior swabs to detect respiratory viruses.

Our novel ANS method has some limitations. First, PCR inhibition occurred in some samples. Whilst inhibition occurs infrequently in PCR testing, the frequency was higher than expected. We suspect the inhibition was likely attributable to the 3-D printing material used in this study for the “small” and “regular” ANS, as this has not been described in other studies using the production version of the adult sized swab [9]. Second, extra steps were needed to extract the PBS from the ANS. Routinely, CTN swabs are swirled in PBS, however, the shape and size of the flocked area of the ANS swab resulted in greater PBS absorption, which then required vortex and pulse spin to allow sufficient PBS to be available for extraction. These steps required laboratory training and additional handling compared to routine CTN processing. Difficulties with PBS extraction from the ANS may be alleviated by using higher volumes of PBS, or inclusion of a standardised universal transport media in the receptacle. Finally, this study included symptomatic children only, which might select those who have moderate to high viral loads.

## Conclusion

The novel ANS had high positive percentage agreement in detection of respiratory viruses, which was comparable to the current CTN standard of care. In addition, it provided a more comfortable experience and was preferred by children, parents/guardians and nurses. Further research in asymptomatic children and those with specific viruses of interest (e.g. SARS-CoV-2) are needed. The ease of use of this method, with potential for self-collection, provides an alternative to CTN testing by medical or nursing staff detection of respiratory viruses, and may contribute to improved public health and epidemiological surveillance.

## Supplementary Information

**Additional file 1:** **Supplementary Figure 1.** Positive percentage agreement subgroup analysis. **Supplementary Figure 2.** Negative percentage agreement subgroup analysis. **Supplementary Figure 3.** Participant flow diagram.

## Data Availability

The datasets used and/or analysed during the current study available from the corresponding author on reasonable request.

## Abbreviations

ANS: Anterior Nasal Swab
COVID-19: Coronavirus disease 2019
CTN: Combined Throat and Nose Swab
RT-PCR: Reverse transcription polymerase chain reaction
SARS-CoV-2: Severe acute respiratory syndrome coronavirus 2
