# Supplementary material for: A novel anterior nasal swab to detect respiratory viruses: a prospective study of diagnostic accuracy
Source: BMC Pediatr. 2023 Apr 28;23:201. doi: 10.1186/s12887-023-03976-5 (PMC10139914; doi:10.1186/s12887-023-03976-5)
Supplement: Supplementary file 1 — Additional file 1: Supplementary Figure 1. Positive percentage agreement subgroup analysis. Supplementary Figure 2. Negative percentage agreement subgroup analysis. Supplementary Figure 3. Participant flow diagram. [file 12887_2023_3976_MOESM1_ESM.docx]

**Supplementary Figures**

**Supplementary Figure 1**: Positive percentage agreement subgroup analysis

**
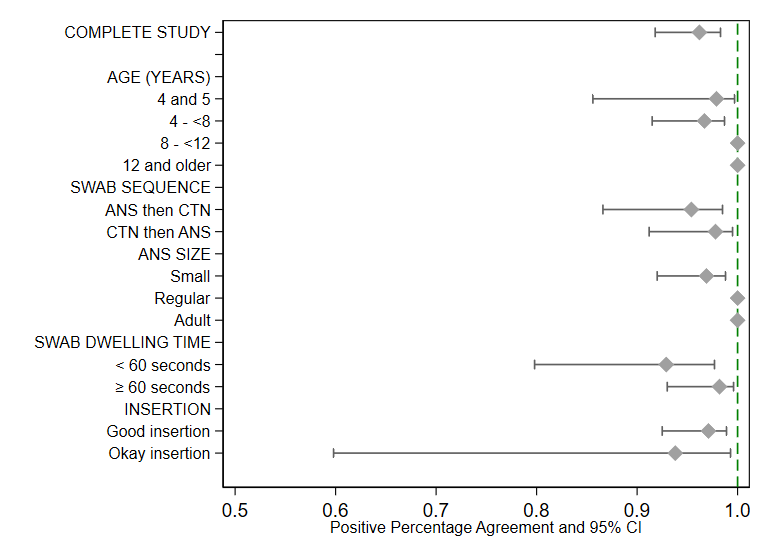
**

**Supplementary Figure 2:** Negative percentage agreement subgroup analysis

**
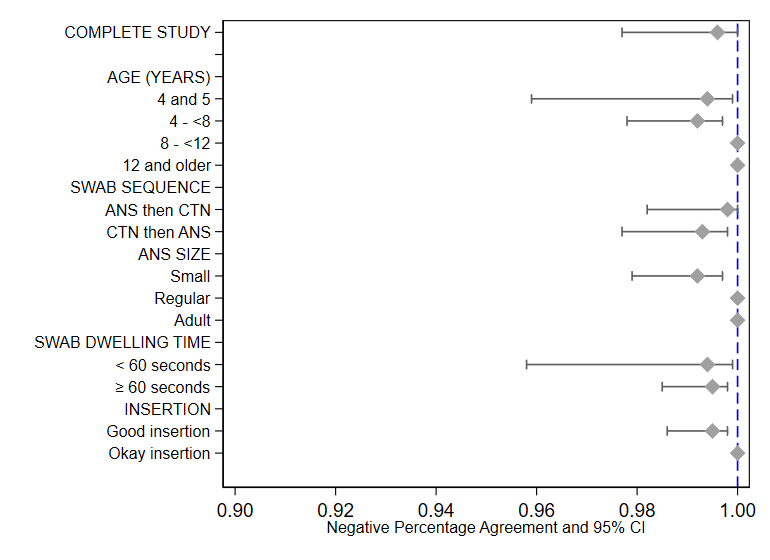
**

**Supplementary Figure 3:** Participant flow diagram

**
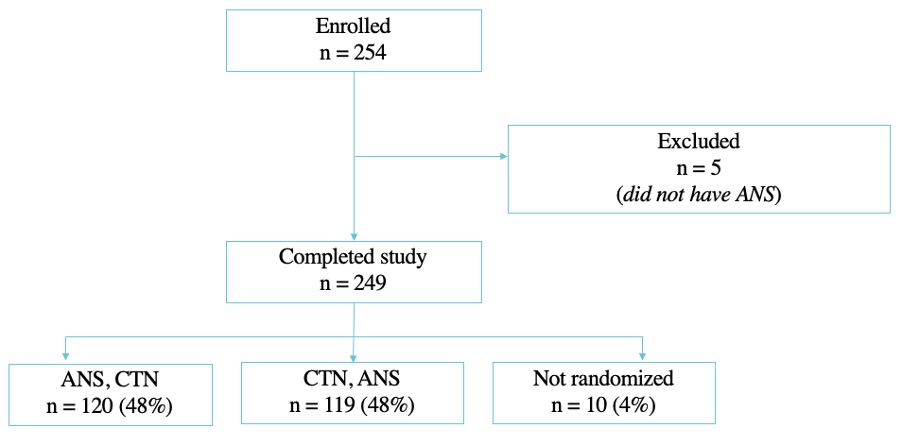
**
